# Breast cancer in women using digoxin: tumor characteristics and relapse risk

**DOI:** 10.1186/bcr3386

**Published:** 2013-02-19

**Authors:** Robert J Biggar, Elisabeth W Andersen, Niels Kroman, Jan Wohlfahrt, Mads Melbye

**Affiliations:** 1 Department of Epidemiology Research, Statens Serum Institut, Artillerivej 5, Copenhagen, 2300, Denmark; 2 Institute of Health and Biomedical Innovation, Queensland University of Technology, 60 Musk Avenue, Brisbane, 4059, Australia; 3 Danish Breast Cancer Cooperative Group and Department of Breast Surgery, Rigshospitalet, Blegdamsvej 9, Copenhagen, 2100, Denmark

## Abstract

**Introduction:**

Digoxin use is associated with increased incidence of breast and uterus cancers. We postulated that digoxin use might affect tumor characteristics and increase relapse risk in women with breast cancer.

**Methods:**

Incident breast cancer cases in Danish women (n = 49,312; 1995 to 2008) were identified. Analyses were conducted in women 20 to 74 years old. Relapse hazard ratios (HR) were compared in women using and not using digoxin, adjusting for age, calendar period, protocol, tumor size, nodal involvement, histology grade, estrogen-receptor (ER) status, and anti-estrogen therapy in Cox regression models.

**Results:**

At diagnosis, tumors in digoxin users were more likely ER+ (85.4% vs. 78.6%: *P *= 0.002) and have grade 1 ductal histology (37.2% vs. 25.7%; *P *= 0.004), compared to non-users. 45 relapses occurred in women already using digoxin at breast cancer diagnosis (1,487 person-years); 24 relapses occurred in women later starting digoxin (384 person-years). Overall relapse risk HR in digoxin users was 1.13 (95% confidence interval: 0.88, 1.46) compared to non-users. Relapse risk in digoxin users was significantly increased in the first year (2.19; 1.26, 3.78) but not thereafter (0.99; 0.74, 1.32) (*P *= 0.02 for difference in HRs). First-year relapse hazard was high in digoxin-using women with ER+ tumors (2.51; 1.39, 4.55) but not ER- tumors (0.72; 0.10, 5.27). Recurrence hazard was not significantly changed among digoxin-using women also using tamoxifen.

**Conclusions:**

Breast cancers arising in digoxin-using women had better prognostic features. After adjustment for markers, overall breast cancer relapse risk in digoxin users was not increased significantly, although recurrence hazards for ER+ tumors were higher in the first year following diagnosis.

## Introduction

Digoxin is a phyto-estrogen widely used in the care of patients with heart disease, where its effect is mediated by the Na^+^K^+^-ATPase ion pump [1]. However, it also binds to the estrogen receptor (ER) and appears to be capable of causing gynecomastia [2]. Women currently using digoxin are at 30% to 50% increased risk of developing incident breast [3,4] and uterus [5] cancer compared to non-users, likely through estrogen-mimicking activity [2]. In Denmark, exogenous estrogen use also has been reported to increase relapse risk in women with a history of breast cancer and, in practice, use is contraindicated for this group [6,7]. We therefore examined if clinically relevant tumor characteristics [8] or relapse risk might differ in women who developed breast cancer while currently using digoxin, adjusting for age, calendar year and protocol.

A priori, we predicted that any effects on recurrence would be most apparent in women with ER-positive rather than ER-negative cancers, given that digoxin likely exerts its effect via attachment to the ER [2]. In the past two decades, anti-estrogen therapies have become increasingly used in women with ER-positive tumors and might affect an impact from digoxin. The two major classes of anti-estrogen drugs act by different mechanisms. Tamoxifen and drugs of this class (hereafter referred to as tamoxifen) interfere with ER binding [9] and might diminish any deleterious effects of digoxin respect to recurrence. In contrast, aromatase inhibitors (AIs), now commonly used in postmenopausal women, reduce systemic estrogen levels but would not be expected to affect digoxin-related excess risks [10]. Therefore, we also predicted that any digoxin-related risk of relapse, would be higher in women on AIs than those on tamoxifen [2].

## Materials and methods

Cases were identified through data in the Danish Breast Cancer Cooperative Group [11], which has centrally recorded information on women with breast cancer in Denmark since 1978. Heart disease requiring digoxin use is a serious co-morbid condition affecting overall prognosis. Because we could not confidently determine the precise cause of death, we chose first relapse as our outcome. Among women entering treatment protocols (mostly those with stage 1 and 2), relapse was defined as the first appearance of new tumor (local/regional recurrence, contralateral breast cancer, or metastatic disease). Relapse information was recorded only for women enrolled in protocols, and therefore patients not in protocols were excluded. The current study included women diagnosed from 1 January 1995 and followed patients from diagnosis to first relapse, death, loss to follow-up, or to 31 December 2008, whichever came first. Patient linkage in registry data was undertaken by authorized national registries and the investigators received only anonymous and untraceable grouped data for analysis. Approvals for this study were obtained in advance from the Danish Ethical Review Board and the Danish Data Protection Agency.

Registry information included diagnosis date, age at diagnosis, tumor size, number of positive lymph nodes, histology grading, ER status, and anti-estrogen therapy. We obtained information on digoxin use from the Danish Register of Medicinal Products Statistics [12], a nationwide centralized registry, which has recorded individual prescriptions for pharmaceutical drugs since 1 January 1995. Digoxin is the only digitalis preparation in the Danish formulary (CO1AA05 in the Anatomical Therapeutic Chemical (ATC) classification, available from the World Health Organization Collaborating Centre for Drug Statistics Methodology, Norwegian Institute of Public Health, PO BOX 4404 Nydalen, NO-0304, Oslo, Norway). Postmenopausal use of estrogen-containing preparations has declined since 1995 and is uncommon in women more than 70 years old [13], which is the group most likely to be using digoxin [2].

Evaluation included women using digoxin at breast cancer diagnosis and those who began using digoxin after breast cancer was diagnosed. Biomarkers affecting prognosis (histology, tumor size and nodal involvement) were evaluated among those already using digoxin at breast cancer diagnosis, adjusted for age, calendar year and protocol. Hazard ratios (HRs) for recurrence were assessed in those continuing or starting digoxin after diagnosis. Those starting digoxin after diagnosis were considered unexposed until they started by including digoxin use as a time-dependent variable. Women were considered digoxin users only for the time of current use, considered to be six months after their most recent prescription. Thus, in time-dependent analyses, a woman was reclassified as a non-user six months after a prescription, unless she filled a new prescription. We have previously used this measure, finding that increased breast cancer incidence was only seen during the time of current digoxin use [4].

**Figure 1:**
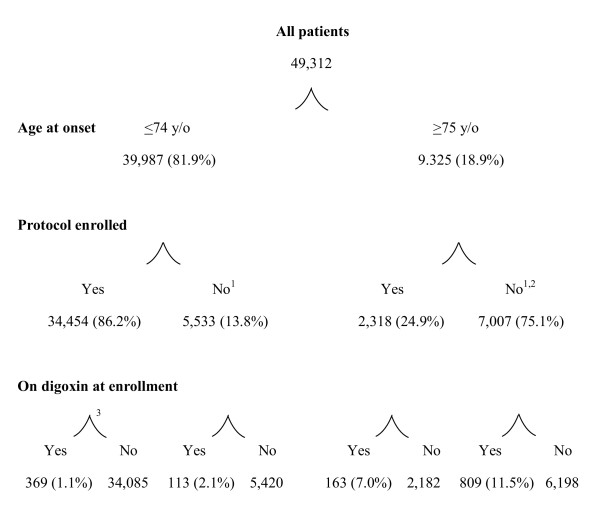
**Women over 20 years old with breast cancer in Denmark, 1995 to 2008**. ^1^Excluded from analysis of relapse because relapse data were not available; ^2^includes 26 patients (none on digoxin) who were assigned to protocol but who accrued no time on protocol treatment; ^3^main analysis file.

Protocol criteria usually exclude women with advanced cancers or complicated co-morbid conditions, since such patients may need individualized care outside standard guidelines. Additionally, older or ill women were more likely to refuse protocol therapy. Due to missing relapse information, patients not in protocols could not be used. We examined demographic and tumor characteristics at breast cancer diagnosis by age. Selecting age <74 years provided the maximum inclusion of cases with protocol-accessible data (Figure 1). As was done in previous studies [4,5], we examined HRs in women currently using angina drugs but not digoxin, as a comparison for breast cancer risk in women with heart disease. Angina-drug exposure was treated as a time-dependent variable in which digoxin users were excluded (in other words, angina drug users compared to neither angina nor digoxin users), as previously defined [4]. The angina drug group included both nitrites and nicorandil (ATC codes: CO1DA02, CO1DA08, CO1DA014, CO1DX16). We previously reported that angina drug use did not affect the incidence of breast [4] or uterus cancer [5]. Angina drugs were started at a slightly younger age than digoxin (median age at first drug use in all women since 1995, 70 and 78 years, respectively [5]).

Exposure to tamoxifen and/or AIs was identified by patient allocation to arms of protocols that included these drugs. Details of drug type, exposure duration and dose were not available by individual. When used, anti-estrogen treatments were typically started within 4 to 8 weeks of diagnosis. The AIs are now commonly used in postmenopausal patients with breast cancer [10], the age group that most commonly uses digoxin. However, AI use did not become widespread until 2005 [10]. Therefore, follow-up time in AI users was limited. Furthermore, many potential users were still enrolled in blinded protocols (individual use unknown) and could not be enrolled in this sub-analysis, further limiting power. In the earlier years of this study, most women using anti-estrogen therapies were taking tamoxifen, regardless of their age. In analysis of the impact of treatment, patients were grouped by those exposed to tamoxifen only, AIs only, and those who took neither (54% of all patients). Patients who received both tamoxifen and AIs at any time during their treatment course (22%), and those still in blinded protocols (3%) were excluded from analysis of the impact of anti-estrogen therapy on the HR for relapse in digoxin users.

Relapse risk was compared in women using digoxin to those not using digoxin by calculating the HR with 95% CIs using Cox regression models [14]. Age profiles differed between digoxin users and non-users. Therefore, the HR for recurrence was adjusted for age at breast cancer diagnosis (20 to 39 years old, and in 5-year age groups thereafter). Six protocols were used during the study time period, each with different criteria (for example, low- or high-risk cancers). We therefore adjusted for protocol. Since therapy improvements likely reduced HRs for relapse during the study period, we also adjusted for calendar year period (1995 to 1999, 2000 to 2004, and 2005 to 2008). Finally we adjusted for tumor size, histology grade, number of positive nodes, and ER status at time of diagnosis (unless being evaluated in analysis).

**Figure 2:**
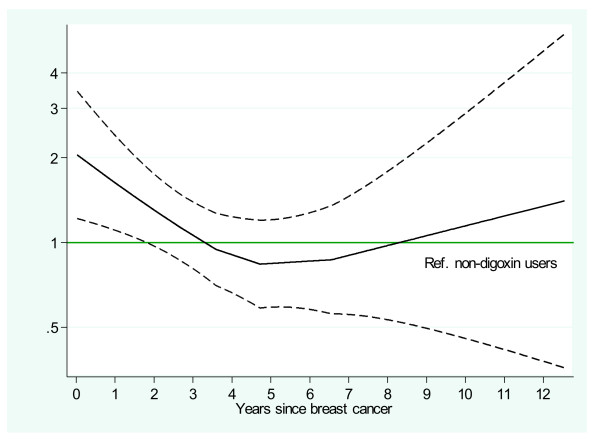
**Estimated relapse hazard ratio (95% CI) for digoxin-exposed compared to unexposed (referent) women with breast cancer, adjusting for current age group and stratifying by protocol, protocol year, histological grade, node status, tumor size, and estrogen receptor status**.

In Figure 2, log-HRs with 95% CIs were estimated from a locally weighted scatterplot smoothed (LOESS)-curve of the scaled Schoenfeld residuals [15] for digoxin exposure for each relapse time. In primary analysis, subjects with missing data were excluded, but in sensitivity analyses, patients with missing data were included with imputation of missing information [16]. The imputation models were ordinal logistic for tumor size, positive nodes and histology grading and included all the variables available at baseline as well as log person-years (py) and relapse status as explanatory variables. Missing information within each variable was assumed to be random, and 10 imputations were included in the model. The distribution of the imputed values was found to be similar to those in the original data.

## Results

We identified 49,312 women with incident breast cancer in the study period, of whom 1,454 (2.9%) used digoxin at breast cancer diagnosis. However, 9,325 patients were >75 years old, among whom the majority (75.1%) were not enrolled in protocol studies (Figure 1) and hence lacked relapse information. In patients >75 years old, digoxin use was more frequent in non-participants (11.5%) than in protocol enrollees (7.0%). Among patients <74 years old, 86.2% participated in protocol studies and tumor characteristics were 97.2% complete (Table 1). In women <74 years old, digoxin use was also more common in non-participants (2.1%) than in participants (1.1%).

**Table 1:** Patients, relapse and follow-up in women with breast cancer by use of digoxin

	Patients, number (%)		*P*-value^1^	Relapse, number		Total person-years of follow-up	
	Digoxin use at breast cancer			Digoxin use at breast cancer		Users of digoxin	Non-users of digoxin
	Yes	No		Yes	No		
**Total**	369 (100)	34,085 (100)		45	5,061	1,487	148,418
							
**Age, years **							
20 to 54	12 (3.2)	13,593 (39.9)	<0.001	2	2,217	68	63,050
55 to 60	36 (9.8)	7,097 (20.8)		5	1,059	165	31,240
61 to 69	163 (44.2)	9,764 (28.6)		20	1,288	641	39,832
70 to 74	158 (42.8)	3,631 (10.7)		18	497	614	14,296
**Year of onset **							
							
1995 to 1999	164 (44.4)	10,612 (31.1)	<0.001	29	2,563	810	64,854
2000 to 2004	111 (30.1)	12,250 (35.9)		12	1,861	512	63,785
2005 to 2008	94 (25.5)	11,223 (32.9)		4	637	165	19,779
**Tumor size**							
No information	5 (1.4)	361 (1.1)	0.602	1	58	13	1,653
							
≤2 cm	226 (62.1)	20,180 (59.8)	0.653	22	2,236	945	90,817
2 to 5 cm	127 (34.9)	12,361 (36.7)		19	2,403	485	51,454
>5 cm	11 (3.0)	1,183 (3.5)		3	364	45	4,495
**Tumor-positive nodes**							
No information	3 (0.8)	45 (0.1)	0.015	0	5	10	96
							
0	216 (59.1)	18,062 (53.1)	0.139	20	1,865	912	82,010
1 to 3	97 (26.5)	10,305 (30.3)		8	1,449	399	45,639
4 to 9	36 (9.8)	3,619 (10.6)		11	977	120	14,095
≥10	17 (4.6)	2,054 (6.0)		6	765	47	6,578
**Histology grading**							
No information	8 (2.2)	608 (1.8)	0.580	2	77	24	2,899
							
Ductal grade I	118 (32.7)	8,602 (25.7)	0.004	13	926	495	40,317
Ductal grade II to III	172 (47.7)	18,652 (55.7)		24	3,199	605	76,710
Non-ductal	71 (19.7)	6,223 (18.6)		6	859	363	28,492
**Estrogen receptor **							
No information	14 (3.8)	1,051 (3.1)	0.446	1	239	57	6,303
							
Positive	303 (85.4)	25,956 (78.6)	0.002	38	3,473	1,262	111,747
Negative	52 (14.6)	7,078 (21.4)		6	1,349	168	30,368

^1^Chi^2 ^tests for comparison of group with no information to those with information, and heterogeneity among groups within strata with known data.

Among 34,454 women <74 years old who contributed time in protocol-treated breast cancer studies in Denmark since 1995, 369 were on digoxin at breast cancer diagnosis (Figure 1). Patients on digoxin at diagnosis had 1,487 py of follow-up (average: 4.0 years; 45 relapses). Additional patients later started digoxin, contributing 384 py of follow-up (24 relapses). In total, during 1,872 py of follow-up while using digoxin, there were 69 relapses. In 34,085 patients not using digoxin at breast cancer diagnosis, there were 148,418 py of follow-up without digoxin use (average: 4.4 years; 4,647 relapses).

Among patients with information available, tumors arising in women who were using digoxin were more likely to be ER-positive (*P *= 0.002) and have grade I ductal histology (*P *= 0.004), and to have less nodal involvement (*P *= 0.14) than among patients not on digoxin. In 33,496 patients (97.2%) with complete covariate information, the overall HR for relapse in women currently using digoxin was not statistically different than in those not using digoxin (HR 1.13, 95% CI 0.88, 1.46), after adjustment for age group, calendar-year of cancer diagnosis, protocol, histology grade, positive nodes, tumor size, and ER status (Table 2). However, in the first year, the HR for relapse was 2.19 (1.26, 3.78), comparing digoxin-users to non-users (Table 3). After the first year, the HR for relapse in patients on digoxin was 0.99 (0.74, 1.32), which was significantly lower than for women using digoxin during the first year (*P *= 0.02). When HRs were permitted to differ between first and subsequent years, digoxin significantly increased the HR for relapse (*P *= 0.04).

**Table 2:** Overall relapse hazard ratios for 33,496 women <74 years old with breast cancer and using digoxin

	Digoxin-exposed	Person-years	Relapse, number	Hazard ratio (95% CI)	*P*-value^1^
**All **	Yes	1,872	69	1.13 (0.88, 1.46)	0.04
	No	148,034	5,037	1.0	
**Estrogen receptor status**					
**Positive **	Yes	1,524	55	1.15 (0.87, 1.52)	0.03
	No	111,485	3,456	1.0	
**Negative**	Yes	280	10	0.93 (0.45, 1.90)	0.94
	No	30,256	1,345	1.0	
**Unknown **	Yes	67	4	1.67 (0.49, 5.69)	0.53
	No	6,293	236	1.0	
**Digoxin exposure with anti-estrogen treatment^2^**					
**Tamoxifen **	Yes	353	26	1.04 (0.68, 1.60)	0.36
	No	23,742	1,431	1.0	
**Aromatase inhibitors **	Yes	79	4	1.87 (0.46, 7.56)	0.68
	No	2,467	87	1.0	
**Neither **	Yes	1,038	33	1.10 (0.75, 1.61)	0.49
	No	81,704	2,815	1.0	

Risk compares patients (complete information only) using digoxin to those not using digoxin (referent) overall and by first and subsequent years after breast cancer diagnosis, by estrogen receptor status and allocated anti-estrogen treatment regimen. ^1^The *P- *value evaluates the overall digoxin effect on the hazard ratios for relapse, permitting different digoxin effects in the first year and later years of follow-up. ^2^Not presented are risks in subjects who switched between tamoxifen and aromatase inhibitors (that is, they were exposed to both at some time in their treatment) and those still in blinded protocols (exposure unknown).

**Table 3:** Hazard ratios for breast cancer relapse by time period of digoxin exposure

		First year			Subsequent years		
	Digoxin-exposed	Person-years	Relapse, number	Hazard ratio (95% CI)	Person-years	Relapse, number	Hazard ratio (95% CI)
**All **	Yes	350	15	2.19 (1.26, 3.78)	1,521	54	0.99 (0.74, 1.32)
	No	30891	699	1,0	11,7143	4,338	1.0
**Estrogen receptor status**							
**Positive **	Yes	285	13	2.51 (1.39, 4.55)	1,239	42	0.98 (0.71, 1.36)
	No	23,524	405	1.0	87,961	3,051	1.0
**Negative **	Yes	51	1	0.72 (0.10, 5.27)	229	9	0.97 (0.45, 2.09)
	No	6,404	262	1.0	23,852	1,083	1
**Unknown **	Yes	15	1	4.29 (0.43, 42.4)	53	3	1.27 (0.29, 5.58)
	No	963	32	1.0	5,330	204	1.0
**Digoxin exposure with anti-estrogen treatment^1^**							
**Tamoxifen **	Yes	71	6	1.90 (0.81, 4.67)	282	20	0.89 (0.54, 1.47)
	No	5,382	185	1.0	18,360	1,246	1.0
**Aromatase inhibitors **	Yes	21	1	2.39 (0.26, 22.4)	58	3	1.63 (0.28, 9.43)
	No	1,037	12	1.0	1,430	75	
**Neither **	Yes	193	8	1.76 (0.74, 4.21)	846	25	1.00 (0.65, 1.54)
	No	16,141	461	1.0	65,563	2.354	1.0

Risk compares patients (complete information only) using digoxin to those not using digoxin (referent) overall and by first and subsequent years after breast cancer diagnosis, by estrogen receptor status and allocated anti-estrogen treatment regimen. ^1^Among tamoxifen and aromatase inhibitor users, hazard ratios for relapse in digoxin users for the first year and and at one year did not differ (*P *= 0.85 and *P *= 0.52, respectively).

Among women with ER-positive breast cancers, 13 relapses occurred in digoxin users during the first year after diagnosis (HR 2.51, 95% CI 1.39, 4.55) (Table 3). In women presenting with ER-negative tumors, only one relapse occurred in digoxin users during the first year. The resulting HR for digoxin use, 0.72 (0.10, 5.27), was low but statistically unstable, and the difference in HR between women with ER-positive and ER-negative tumors was not statistically significant (*P *= 0.24). After the first year, the HR for relapse with digoxin use was similar in women with ER-positive (HR 0.98, 95% CI 0.71, 1.36) and ER-negative (HR 0.97, 95% CI 0.45, 2.09) breast cancers (*P *= 0.98).

To determine if tamoxifen or AI use affected the HR for digoxin-related relapse, we examined anti-estrogen therapy assigned to each ER-positive patient (Table 2). In the small subset of digoxin-using patients who also got a single type of anti-estrogen therapy, relapse occurred in twenty-six patients using tamoxifen only and four patients using AIs only. Among current-digoxin users, the overall HR for relapse in women only exposed to tamoxifen was 1.04 (95% CI 0.68, 1.60), whereas in women only using AIs, the HR was 1.87 (0.46, 7.56). This difference in relapse HRs was not statistically significant (*P *= 0.43).

As a sensitivity analysis of the effect of digoxin on the HR for relapse, we re-analyzed outcomes on all 34,454 protocol-enrolled women, imputing missing data in the 2.8% women with incomplete confounder information and using fully adjusted models. The results were similar to those in the main analysis. In the first year after diagnosis, the HR for relapse was 2.22 (95% CI 1.30, 3.77). For those exposed in periods after the first year following diagnosis, the HR was 0.98 (0.74, 1.30) among current digoxin-users. Thus, missing data did not affect our findings.

In a further sensitivity analysis, we also re-analyzed the dataset of protocol-enrolled women with complete confounder information using patients of all ages and applying the same adjustments. The patterns were again similar. Among 1,077 women currently using digoxin, 81 relapses occurred during 2,191 py of follow-up. After complete adjustment, the overall HR among women currently using digoxin was 1.15 (95% CI 0.91, 1.46), compared to the HR for those using neither drug. In the first year, the HR for relapse was 2.40 (95% CI 1.52, 3.77). For digoxin exposures more than one year after diagnosis, the HR for relapse was 0.95 (0.72, 1.26), without significant variation in the HR by years after diagnosis in this period. Thus, the age restriction did not affect the findings.

Finally, we analyzed the HR for relapse among women <74 years old who were using angina drugs but not digoxin. After adjustment for all variables used in the digoxin analyses, the overall HR was 1.40 (95% CI 1.11, 1.75). The HR for relapse in the first year was 1.40 (0.71, 2.74) and for following years, the HR was also 1.40 (1.10, 1.78).

## Discussion

Women taking digoxin had an increased proportion of ER-positive low-grade tumors and less nodal involvement compared to those not taking digoxin at the time of diagnosis. While there was no statistically significant difference in overall risk of relapse for women taking vs. not taking digoxin for the entire follow-up period, recurrence risk was statistically significantly increased (HR 2.2) in the first year after cancer diagnosis in women taking digoxin. Although statistically different from the HR for relapse in digoxin users during subsequent years, the increased risk of recurrence in the first year could just be an unusual chance finding.

Thus, the recurrence risk patterns for digoxin use are similar to those reported for postmenopausal hormone replacement therapy, which is also associated with an increase in breast cancer incidence [17,18]. Specifically, women who develop breast cancer while on estrogens also have a higher proportion of ER-positive better-prognosis tumors [8] and those who continue or are placed on estrogens also have an increased recurrent risk [6,7]. Use of progestin-containing hormonal combinations has been proposed to increase risk of recurrence more than estrogen alone [19]. In this regard, the pharmacology of digoxin would resemble that of estrogen-alone use, assuming it acts as an estrogen-agonist. Long-term survival appears to be better in those who develop breast cancer while using estrogen [20-23], perhaps because the tumors have better prognosis characteristics, but we did not examine survival in digoxin-users because of concern about the impact of co-morbidity from cardiac conditions.

An objective of this study was to examine the effect of anti-estrogen therapy on the effects of digoxin. Use of tamoxifen reduces progression of ER-positive ductal carcinoma *in situ *[9,24]. If digoxin mediates its effect through the ER, then blocking the ER with tamoxifen might interfere with a digoxin effect. In contrast, AI acts by blocking estrogen production, leaving the ERs intact and digoxin without competition for attachment to the ER [2]. The modest changes in HR with tamoxifen or AI use are possibly consistent with mediation of a digoxin effect through the ER, but we acknowledge that these results were not statistically significant and require further evaluation. A further consideration is whether digoxin acts via interaction with the ER or via an interaction with the Na^+^K^+^-ATPase pump, the major target of digoxin activity [1]. The observation that changes in risk are specific to ER-positive tumors argues for a digoxin effect mediated via ER interaction.

The strengths of this study are its large size, population-representativeness, and completeness. A high proportion of women <74 years old were enrolled in protocols and the modest losses due to exclusion did not influence outcomes. High quality data were available about the patients, their tumors, the therapies they received, and recurrence outcomes. During this period, there was no awareness of the potential risks of digoxin use during this period, and therefore digoxin use would not be related to the cancer treatment regimen. Studies of anti-estrogen therapies were restricted to patients with ER-positive tumors who were randomly selected within the protocol assignment to receive them. They were therefore unbiased by the indication for anti-estrogen therapy. Being under observation for cardiac problems might accelerate the recognition of relapse. However, all women in their first year after entering therapy for breast cancer will be under close scrutiny for relapse, regardless of their cardiac condition, and a two-fold HR increase seems unlikely.

We also evaluated risk of relapse in women using vasodilating drugs but not digoxin, to be sure that women with cardiac conditions did not have exceptional risk of relapse for reasons other than digoxin use. To our surprise, women using vasodilators were at significantly increased risk of relapse in both the first year and subsequent years of follow-up. Vasodilators have no estrogen-mimicking properties, are not associated with gynecomastia, and do not increase the incidence of breast cancer [4]. Women with cardiac conditions might have received other drugs that induce gynecomastia, which we have speculated might increase breast cancer incidence [2]. We acknowledge that unknown confounders might have influenced the associations observed for both digoxin and angina drugs.

The association of relapse with digoxin and angina drugs potentially could suggest that risk might be attributable to heart disease, possibly mediated through obesity. In our study, measures of obesity were not available. Postmenopausal breast cancer incidence is high in obese women [25,26]. The cancers are especially likely to be ER-positive, and the route is thought to be related to higher levels of estrogen, which are also high in older obese women [27]. Older obese women are also at increased risk of cancer diagnosis in the contralateral breast [27,28]. Similarly, there is ample evidence that obese women have poorer survival rates following ER-positive breast cancer [29-32]). However, obesity is associated with increased mortality regardless of coincident breast cancer [33].

The effect of obesity on risk of recurrence is unclear, but in those studies reporting differences, the risk in normal-weight and obese women begins to diverge at only 3 to 4 years following treatment [29,31,32]. Whether these recurrences represent new cancers or true relapses is unknown. Obesity is not associated with increased risk of relapse in the first year after diagnosis, the period in which we observed an increased HR with digoxin use. Thus, we cannot attribute the two-fold increased HR for relapse in the first year to an interaction with obesity. However, we cannot exclude confounding from another unknown co-factor associated with heart disease.

## Conclusions

While breast cancer incidence is increased in the women taking digoxin [3,4], the breast cancers occurring in women taking digoxin had better prognostic features than in women not using digoxin. After adjustment for prognostic features, women who continued to use digoxin after breast cancer onset had a significantly higher risk of recurrence in the first year but not overall vs. those not using digoxin.

## Abbreviations

AI: aromatase inhibitor; ATC: anatomical therapeutic chemicals; ER: estrogen receptor; HR: hazard ratio; LOESS: locally weighted scatterplot smoothing; py: person-years; n: number of observations.

## Competing interests

The authors declare that they have no competing interests.

## Authors' contributions

RB conceived of and organized the study and was primarily responsible for drafting the manuscript; EA analyzed the data; NK collected primary data and provided clinical input; JW obtained the dataset and guided statistical analysis; MM helped with its design and coordination. All authors assisted with drafting the manuscript and approved the final manuscript.
